# Supplementary material for: Oropharyngeal Carriage of hpl-Containing Haemophilus haemolyticus Predicts Lower Prevalence and Density of NTHi Colonisation in Healthy Adults
Source: Pathogens. 2021 May 10;10(5):577. doi: 10.3390/pathogens10050577 (PMC8151607; doi:10.3390/pathogens10050577)
Supplement: Supplementary file 1 [file pathogens-10-00577-s001.zip › pathogens-1199647-supplementary.pdf]

SUPPLEMENTAL INFORMATION

Table S1| Oropharyngeal colonisation frequencies of NTHi and Hh strains among 257 healthy adults in Australia.

	Colonisation frequency (%)	
**Colonisation status**	**Adults (18-65 years)**	**Elderly (>65 years)**
**Total Hh-*hpl*$^{-}$**	**63/184 (34)**	**23/73 (32)**
NTHi+	39/63 (62)	21/23 (91)
NTHi-	24/63 (38)	2/23 (9)
**Total Hh-*hpl*$^{+}$ (non-predominant [a])**	**40/184 (22)**	**7/73 (10)**
NTHi+	10/40 (25)	1/7 (14)
NTHi-	30/40 (65)	6/7 (86)
**Total Hh-*hpl*$^{+}$ (predominant [b])**	**39/184 (21)**	**8/73 (11)**
NTHi+	5/39 (13)	0/8 (0)
NTHi-	34/39 (87)	8/8 (100)
**Total Hh+**	**142/184 (77)**	**38/73 (52)**
**Total Hh-**	**42/184 (23)**	**35/73 (48)**
**Total NTHi+**	**54/184 (29)**	**39/73 (53)**

$^{+/-}$ Detection by PCR for corresponding gene targets *siaT* (NTHi), *hypD* (Hh) and the *hpl* ORF; **[a]** Hh-*hpl*$^{+}$ is not the predominant Hh genotype (<0.5 of total Hh); **[b]** Hh-*hpl*$^{+}$ is the predominant Hh genotype (>0.5 of total Hh)

Table S2| Average oropharyngeal colonisation density of NTHi and Hh strains among 257 healthy adults in Australia.

	Average density of NTHi and Hh genotypes (GE) (95% CI)		
**Age group**	**NTHi**	**Hh-*hpl*$^{-}$**	**Hh-*hpl*$^{+}$**
**Adults**	3.08×10^{5} (1.82×10^{5} -6.65×10^{5})	2.25×10^{5} (1.13×10^{5} – 3.67×10^{5})	1.62×10^{5} (7.54×10^{4} – 3.09×10^{5})
**Elderly**	6.84×10^{4} (3.18×10^{4} – 1.30×10^{5})	1.89×10^{4} (1.05×10^{4} – 8.87×10^{4})	3.48×10^{4} (1.97×10^{3} - 8.12×10^{4})

S1. Triplex real-time PCR assay design, optimisation and validation

A triplex PCR assay was designed to detect and quantify Hi, Hh and the *hpl* ORF. The targets used for discrimination of Hh (*HypD*) and NTHi (*SiaT*) have previously been described and validated [1]. For detection of the *hpl* ORF, primers were designed using the ORF of the model Hpl-producer, Hh strain BW1 (GenBank MN720274) [2]. Primer specificity was confirmed using discontiguous megaBLAST analysis performed across 115 complete and 862 draft genome assemblies for *Haemophilus.spp* available from Genbank. A nonredundant nucleotide (nr/nt) collection BLAST search was also conducted to determine amplicon specificity in non-*Haemophilus* genomes. Following optimisation in singleplex format, the three assays were merged into triplex

format. Annealing temperature was optimised using an 8-step temperature gradient ranging from 53-63°C. Specificity of amplicons was determined by gel electrophoresis and the optimal temperature was selected based on highest yield of amplicons of the correct size in the absence of non-specific amplification. PCR specificity for Hi, Hh and *hpl* was determined using 13 Hh strains with varying *HPL* sequence similarity to BW1, 13 Hi strains and 9 other genera representing common upper respiratory tract flora. Reaction efficiency of triplex reaction was determined using 10-fold dilutions of control strains over the range of 2 to 2×10^{-8} ng (Hi ATCC49247, Hh ATCC 33390 and Hh BW1). Limits of quantification (LoQ) values were determined for *hypD* and *siaT* targets in triplex format based on criteria where replicates at a given dilution with a cycles to threshold (Ct) standard deviation (σ) of ≥0.8 were considered to exceed the LoQ, with one or more amplification failures also deemed a LoQ failure. The upper LoQ value was not determined due to the unlikelihood of encountering such high DNA concentrations in clinical specimens. The lower limit of detection was also determined for the *hpl* target and defined from linearity data (Figure S1).

In silico specificity for the *HPL* amplicon revealed 97-100% primer and probe nucleotide sequence identity for 26 of 61 complete or draft Hh genomes available in Genbank. Sequence similarity to the *HPL* amplicon was detected in 20 *H. influenzae* genomes out of 757 complete and draft genome assemblies. However, all alignments contained a minimum of 4 SNPs in the reverse primer and Taqman probe and did not contain any sequence homology with the forward primer. *hpl* was also detected in 3 genome assemblies available for *Haemopilus quentini*, which was expected based on previous analysis of the *hpl* ORF [2]. Despite close relatedness to Hh, isolation of this strain has only been described in the genitourinary tract so is unlikely to be co-isolated from respiratory specimens [4]. Comparison of 11 previously sequenced *hpl* ORFs [2] to these databases yielded the same results, indicating high sensitivity to known *hpl* sequence variants (ranging from 85-100% homology to BW1). PCR of these isolates, and additional Hh, Hi and common upper respiratory tract colonisers confirmed specificity for each target.

Table S3| Summary of primer and LNA-probe sequences, and expected amplicon size for the *hypD*, *siaT* and *hpl* targets.

Primers and Probes	Sequence	Amplicon Size (bp)
**hypD Forward**	5'- GGCAATCAGATGGTTTACAACG	187
**hypD Reverse**	5'- CAGCTTAAAGYAAGYAGTGAATG	
**hypD LNA-probe**	/5HEX/CCA+C+AA+C+GA+G+AATTAG/3IABkFQ/	
**siaT Forward**	5'- AATGCGTGATGCTGGTTATGAC	138
**siaT Reverse**	5'- AATGCGTGATGCTGGTTATGAC	
**siaT LNA-probe**	/56-FAM/A+GA+A+GCAGC+A+G+TAATT/3IABkFQ/	
**HPL Forward**	5'- TATTCCTAATGATCCCGCT	120
**HPL Reverse**	5' - TCTTTTTTCGCTACCCCT	
**HPL LNA-probe**	/5Cy5/AT+CCATTTA+TCGG+CACGTTCT/3IAbRQSp/	

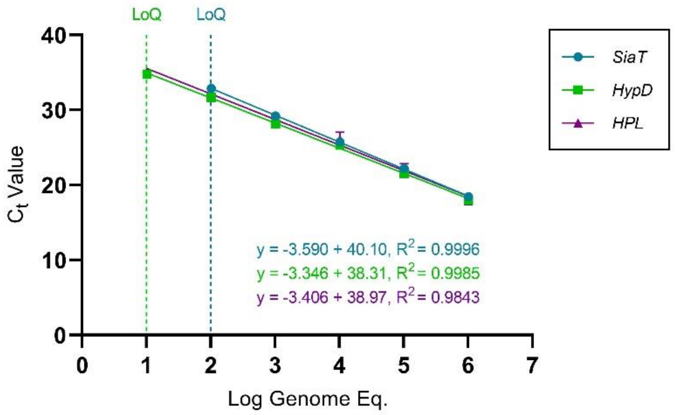

Figure S1 |PCR efficiency and measure of LoQ and LoD. Shows PCR C_t values for the *SiaT* ,*HypD* and *HPL* targets measured from serial dilutions of Hi, Hh^{HPL-} and Hh^{HPL+} control strain DNA over the range of 2 to 2 × 10-8 ng. Data points are represented as mean +/- SEM of two separate experiments, each conducted in duplicate.

Table S4| Genotypic and phenotypic characteristics of Hh strains containing the *hpl* ORF. Sequences were determined by whole-genome sequencing, performed using the MiSeq (Illumina) platform. ORF similarities are compared to a model Hpl-producing strain (Hh-BW1) that has demonstrated high anti-NTHi activity *in vitro.*

Hh Strain	% Identity of the *hpl* ORF	Hpl production
**BW1**	-	+
**BW5**	99% (807/819)	+
**BW15**	100% (819/819)	+
**BW18**	100% (819/819)	+
**BW36**	100% (819/819)	+
**CF14**	96% (786/819)	+
**L19**	85% (709/833)	-
**L117**	85% (709/833)	-
**L152**	85% (709/833)	-
**L153**	98% (805/819)	+
**NF4**	100% (819/819)	-
**NF5**	100% (819/819)	+
**NF6**	96% (786/819)	+
**NF1**	80% (636/794)	-
**RHH122**	100% (819/819)	+
**L37**	80% (636/794)	-
**CF26**	79% (630/794)	-
**L52**	98% (805/819)	+
**L56**	81% (621/768)	-
**OP1**	100% (819/819)	+
**OP2**	100% (819/819)	+
**OP3**	100% (819/819)	+
**OP4**	100% (819/819)	+
**NF11**	96% (788/819)	+

$^{+/-}$ Hpl-producing/non-producing (determined by detection of *hpl* expression and bioassay of culture supernatants).

References

[1] Price EP, Harris TM, Spargo J, Nosworthy E, Beissbarth J, Chang AB, et al. Simultaneous identification of Haemophilus influenzae and Haemophilus haemolyticus using real-time PCR. Future Microbiology. 2017.
[2] Latham RD, Torrado M, Atto B, Walshe JL, Wilson R, Guss JM, et al. A heme-binding protein produced by Haemophilus haemolyticus inhibits non-typeable Haemophilus influenzae. Molecular Microbiology, In press. 2019:626416.
[3] Waiblinger H-U, Grohmann L. Guidelines for validation of DNA extraction methods applied in subsequent PCR analysis of food and feed products for the presence of genetically modified material. Journal für Verbraucherschutz und Lebensmittelsicherheit. 2014;9:183-90.
[4] Glover WA, Suarez CJ, Clarridge III J. Genotypic and phenotypic characterization and clinical significance of 'Haemophilus quentini'isolated from the urinary tract of adult men. Journal of medical microbiology. 2011;60:1689-92.
